# Neurogenesis From Neural Crest Cells: Molecular Mechanisms in the Formation of Cranial Nerves and Ganglia

**DOI:** 10.3389/fcell.2020.00635

**Published:** 2020-08-07

**Authors:** Karla Méndez-Maldonado, Guillermo A. Vega-López, Manuel J. Aybar, Iván Velasco

**Affiliations:** ^1^Instituto de Fisiología Celular – Neurociencias, Universidad Nacional Autónoma de México, Ciudad de México, Mexico; ^2^Departamento de Fisiología y Farmacología, Facultad de Medicina Veterinaria y Zootecnia, Universidad Nacional Autónoma de México, Ciudad de México, Mexico; ^3^Instituto Superior de Investigaciones Biológicas (INSIBIO, CONICET-UNT), San Miguel de Tucumán, Argentina; ^4^Instituto de Biología “Dr. Francisco D. Barbieri”, Facultad de Bioquímica, Química y Farmacia, Universidad Nacional de Tucumán, San Miguel de Tucumán, Argentina; ^5^Laboratorio de Reprogramación Celular, Instituto Nacional de Neurología y Neurocirugía “Manuel Velasco Suárez”, Ciudad de México, Mexico

**Keywords:** cranial nerve, peripheral nervous system, hindbrain, cell signaling, transcriptional regulatory network, trigeminal nerve, facial nerve, vagus nerve

## Abstract

The neural crest (NC) is a transient multipotent cell population that originates in the dorsal neural tube. Cells of the NC are highly migratory, as they travel considerable distances through the body to reach their final sites. Derivatives of the NC are neurons and glia of the peripheral nervous system (PNS) and the enteric nervous system as well as non-neural cells. Different signaling pathways triggered by Bone Morphogenetic Proteins (BMPs), Fibroblast Growth Factors (FGFs), Wnt proteins, Notch ligands, retinoic acid (RA), and Receptor Tyrosine Kinases (RTKs) participate in the processes of induction, specification, cell migration and neural differentiation of the NC. A specific set of signaling pathways and transcription factors are initially expressed in the neural plate border and then in the NC cell precursors to the formation of cranial nerves. The molecular mechanisms of control during embryonic development have been gradually elucidated, pointing to an important role of transcriptional regulators when neural differentiation occurs. However, some of these proteins have an important participation in malformations of the cranial portion and their mutation results in aberrant neurogenesis. This review aims to give an overview of the role of cell signaling and of the function of transcription factors involved in the specification of ganglia precursors and neurogenesis to form the NC-derived cranial nerves during organogenesis.

## Introduction

**FIGURE 1:**
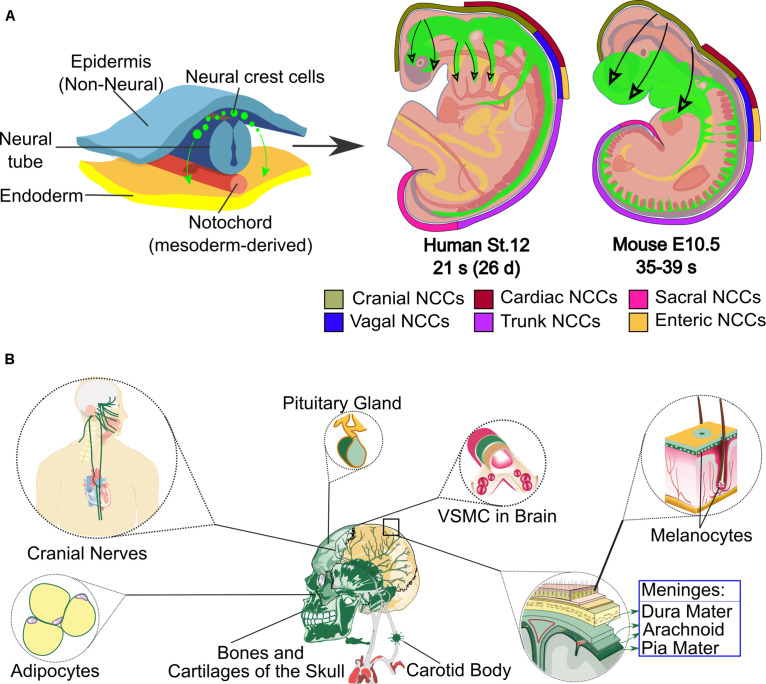
Neural crest origin, regions in human and mouse embryos and some of its cranial derivatives. **(A)** The top-left part of the scheme shows the origin of the neural crest cells (green) that migrate through the embryo. On the top-right side, the level of axial origin (see axial color key) of different regions of the neural crest is represented in developing mouse or equivalent human embryos; the migration of neural crest is represented in green inside the embryos and the direction of migration is marked with black arrows. Depending on their axial level of origin and migratory pathways, neural crest cells adopt different fates and contribute to various tissues and organs. **(B)** The main cranial derivatives, labeled in green, are shown. Abbreviations: d, days, E, mouse embryonic stage; NCCs, Neural Crest Cells; s, somite; St, human stage; VSMC, vascular smooth muscle cells.

During the embryonic development of vertebrates, one of the main events after the gastrulation process is neurulation, which allows the formation of the neural tube (NT). The neural ectoderm generates not only the central nervous system (CNS) but also another set of cells between the NT and the non-neural ectoderm located in the most dorsal part of the NT, called the neural crest (NC) (Hall, 2008; Simões-Costa et al., 2015). This versatile and plastic cell population was first described by Wilhelm His 150 years ago (Hall, 1999). The NC is one of the most important features that separate vertebrates from other chordate organisms. It arises at the posterior and lateral borders of the neural and non-neural ectoderm, the neural plate border (Figure 1) (Cerrizuela et al., 2020).

NC cells (NCCs) are multipotent and give rise to several cell types, depending on the site of origin along the anteroposterior axis of the embryo. NCCs are divided into cranial, trunk (including cardiac), vagal and sacral (Figure 1A) (Minoux and Rijli, 2010; Simões-Costa and Bronner, 2013; Vega-Lopez et al., 2018). Cranial nerves (CN) transmit sensory and motor information between the brain and tissues of the head and cervical region. The CN are formed from the contribution of two specialized embryonic cell populations, cranial NC and ectodermal placodes.

### Origin of the Neural Crest

**FIGURE 2:**
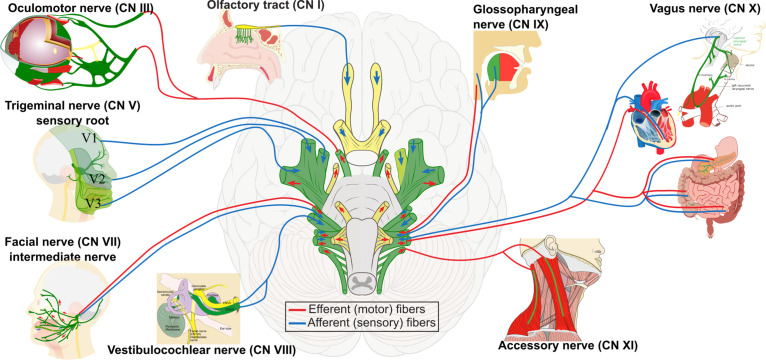
Contribution of neural crest cells to the formation of cranial nerves I, III, V, VII, VIII, IX, X, and XI. These selected cranial nerves are formed by the contribution of cranial placodes and neural crest cells, indicated in green. Neural crest-derived Schwann cells produce peripheral myelination of cranial nerves III–XII. The sensory nerves are the olfactory (I), the optic (II), and the vestibulocochlear (VIII). The motor nerves are the oculomotor (III), the trochlear (IV), the abducens (VI), and the accessory (XI). The remaining are mixed nerves.

**FIGURE 3:**
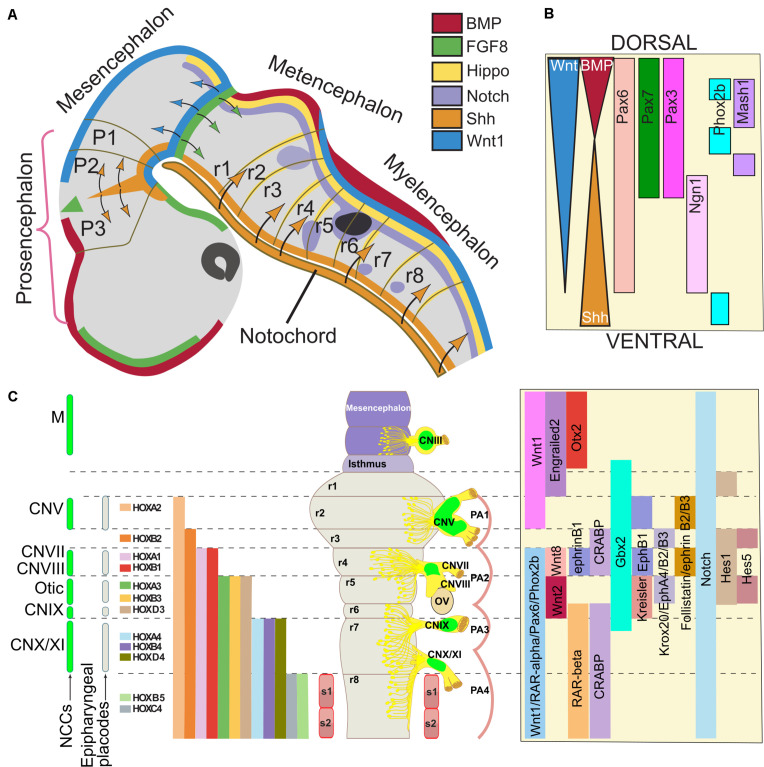
Gene regulatory network involved in neural crest contribution to the formation of cranial nerves. The cranial ganglia and cranial nerves are formed in precise positions along the dorso-ventral and antero-posterior axes of the midbrain/hindbrain region. **(A)** The drawing represents a human embryo at stage 13 (30 days, 32 somites), equivalent to mouse day 9.5-10 (E9.5-10, 20 somites) and chick stage 14 (50 h, 22 somites). The cell signaling pathways that provide developmental cues to neural crest precursors are color-coded; when these factors diffuse, the target regions are indicated with arrows with the same color. In panel **(B)**, an idealized scheme of the hindbrain shows the cell signaling gradients and the genes that establish the dorsoventral pattern. **(C)** The illustration of the human (33 days, stage 14) and chick (stage 21) hindbrain rendered flat to eliminate cerebral flexures. The levels of origin of the neural crest cells (NCCs) and placodes, which contribute to the formation on cranial nerves, are indicated on the left. NCCs from the corresponding rhombomeres also populate other embryo structures in a segmental fashion and generate different craniofacial derivatives. The positions of the cranial ganglia and the otic vesicles are indicated on the right side; the contribution of NCCs is indicated in green. The segmental nested expression of *HOX* genes is color-coded. On the right, signaling pathways and the expression of transcription factors involved in cranial nerve (CN) formation are indicated. Adapted from Lumsden and Keynes (1989), Noden (1991), Yamamoto and Schwarting (1991), Bally-Cuif and Wassef (1995), Takahashi and Osumi (2002), and Müller and O’Rahilly (2011). Abbreviations: CN, cranial nerve; FP, floor plate; M, mesencephalon; NCCs, neural crest cells; OV, otic vesicle; r, rhombomere; PA, pharyngeal arches.

NCCs, which are multipotent, delaminate from their origin and migrate throughout the body to differentiate into several cell types including cells of the peripheral nervous system (PNS), melanocytes, cranial cartilage and bone, neuroendocrine cells, and several other phenotypes (Figure 1B). In humans, at least 47 cell types have been defined as NC derivatives (Vickaryous and Hall, 2006). Proper NC migration relies on environmental cues such as Eph-Ephrins (Smith et al., 1997), Semaphorin-3F (Gammill et al., 2007), Versican (Szabó et al., 2016), the chemokine Stromal cell-derived factor 1 (Theveneau et al., 2013) or Robo2 (Shiau et al., 2008). The migration patterns of NCCs have been clearly described for model organisms like birds, frogs and mice. In all vertebrates, cranial NCCs emerge from the forebrain, midbrain and hindbrain regions (Couly and Le Douarin, 1987; Serbedzija et al., 1992). Depending on their axial origin, cranial NCCs will either migrate through the facial mesenchyme and into the frontonasal process, or will populate the branchial arches (Noden, 1975; Lumsden et al., 1991; Serbedzija et al., 1992). The sensory module of the PNS in the cranial region is composed of an array of paired ganglia adjacent to the hindbrain that transduce the perception of touch, pain, temperature, position and special sensory information from the periphery to the CNS. Cranial NCCs migrate to form sensory ganglia such as the trigeminal (V), the facial (VII), the glossopharyngeal (IX), the vagus (X) CN, and also to form the motor ganglia for the oculomotor (III) and accesory (XI) CN (Table 1 and Figures 2, 3).

**TABLE 1:** Contributions of neural crest cells and placodes to ganglia and cranial nerves.

**Cranial nerve**	**Ganglion and type**	**Origin of neurons**	**References**
CNI – Olfactory (Ensheating glia of Olfactory nerves)		Telencephalon/olfactory placode; NCCs at forebrain	Boyd et al., 2003; Muller et al., 2004; O’Rahilly and Müller, 2007; Barraud et al., 2010
CNIII – Oculomotor (m)	Ciliary, visceral efferent	NCCs at forebrain-midbrain junction (caudal diencephalon and the anterior mesencephalon)	Noden, 1978; Couly et al., 1993; Wahl et al., 1994; Lee et al., 2003
CNV – Trigeminal (mix)	Trigeminal, general afferent	NCCs at forebrain-midbrain junction (from r2 into 1st PA), trigeminal placode	d’Amico-Martel and Noden, 1980; Forbes and Welt, 1981; D’amico-Martel and Noden, 1983
CNVII – Facial (mix)	-Superior, general and special afferent -Inferior: geniculate, general and special afferent -Sphenopalatine, visceral efferent -Submandibular, visceral efferent	-Hindbrain NCCs (from r4 into 2nd PA), 1st epibranchial placode -1st epibranchial placode (geniculate) -Hindbrain NCCs (2nd PA) -Hindbrain NCCs (2nd PA)	D’amico-Martel and Noden, 1983; Lumsden et al., 1991; Barlow and Northcutt, 1997; Begbie and Graham, 2001
CNVIII – Vestibulocochlear (s)	Acoustic: cochlear, special afferent; and Vestibular, special afferent	Otic placode and hindbrain (from r4) NCCs	Barlow, 2002; Krimm, 2007; Sandell et al., 2014
CNIX – Glossopharyngeal (mix)	-Superior, general and special afferent -Inferior, petrosal, general and special afferent -Otic, visceral efferent	-Hindbrain NCCs (from r6 into 3rd PA) -2nd epibranchial placode (petrosal) -Hindbrain NCCs (from r6 into 3rd PA)	Narayanan and Narayanan, 1980; D’amico-Martel and Noden, 1983; O’Rahilly and Müller, 1984; Barlow and Northcutt, 1997
CNX – Vagus (mix) Superior laryngeal branch; and recurrent laryngeal branch	-Superior, general afferent -Inferior: nodose, general and special afferent -Vagal: parasympathetic, visceral efferent	-Hindbrain NCCs (from r7-r8 to 4th and 6th PA) -Hindbrain NCCs (4th and 6th PA); 3rd (nodose) and 4th epibranchial placodes -Hindbrain NCCs (4th and 6th PA)	Narayanan and Narayanan, 1980; D’amico-Martel and Noden, 1983
CNXI – Accessory (m)	No ganglion*	Hindbrain (from r7-r8 to PA 4); NCCs (4th PA)	Muller and O’Rahilly, 1980; O’Rahilly and Müller, 2007

Abbreviations: CN, Cranial Nerve; m, purely motor nerve; mix, mixed nerve (sensory and motor); NC, neural crest; PA, pharyngeal (branchial) arch; r, rhombomere; s, purely sensory nerve. *There is no known ganglion of the accessory nerve. The cranial part of the accessory nerve sends occasional branches to the superior ganglion of the vagus nerve.

NC formation is a complex and multistep process initially directed by cell signaling molecules including Bone Morphogenetic Proteins (BMPs), Wnts (Wingless and Int-1), Fibroblast Growth Factor (FGF), and retinoic acid (RA). These signals reveal the tissue interactions into the ectodermal cell populations, the neural plate, the non-neural ectoderm, and the underlying mesoderm in a highly coordinated manner (Vega-Lopez et al., 2017). It has been proposed that NC specification occurs during gastrulation as a consequence of the action of two successive gradients of secreted signals. A combination of intermediate levels of activity of BMP and Wnt signaling acting on the ectoderm to induce and specify NC precursors at the neural plate border, and a subsequent requirement of both signals is needed for maintenance of specification during neurulation (Aybar and Mayor, 2002; Steventon et al., 2009). In chick embryos, it was shown that NCCs are specified as early as the blastula stage (Prasad et al., 2020). It was demonstrated that, during gastrulation, *Pax7* expression is restricted to cells located in a region in the medial epiblast, which are NC-fated and contribute to the neural folds and later to migrating NCCs (Basch et al., 2006). The inhibition of Pax7 function in chicks inhibited the expression of key NC markers such as *Snai2* (OMIM 602150), *Sox9* (Sry-box 9, OMIM 608160), *Sox10* (OMIM 602229), and *HNK1* (beta-1,3-glucuronyltransferase 1 like, OMIM 151290) (Basch and Bronner-Fraser, 2006). This evidence suggests that the neural plate-prospective ectoderm interaction at the neural plate border might not be a requisite for NC specification or induction, and that neural plate border formation and NC induction might be separable events.

The various research works carried out to study the origin of NCCs have identified genes organized into a gene regulatory network that participate in and control the induction, specification and differentiation of NC (Simões-Costa et al., 2015). An example of this are the transcription factors involved in induction such as FoxD3 (*Forkhead Box D3*, OMIM 611539), Snai2 and Sox9 (Sauka-Spengler and Bronner-Fraser, 2006). García-Castro and co-workers identified a novel pre-neural border state characterized by early Wnt/β-catenin signaling targets that displayed different responses to BMP and FGF signaling from the neural border genes in human cells (Leung et al., 2016). These pre-border genes *Gbx2* (*Gastrulation brain homeobox 2*, OMIM 601135), *SP5* (OMIM 609391), *Zic3* (OMIM 300265) and *Zeb2* (OMIM 605802) had their induction and peak of expression before the classical neural plate border specifier genes such *Msx1/2 (Muscle segment-related homeobox 1/2*, OMIM 142983/123101), *Pax3/7* (OMIM 606597/167410) and *Zic1* (OMIM 600470). Such specifier genes, together with signaling molecules, direct the expression of NC-specific genes like *AP-2* (OMIM 107580), *FoxD3*, *Snai2, Sox9*, and *Sox10*. Specifiers regulate NC effector genes involved in migration (*Sox9*, *Sox10*, *Cad7*) and differentiation [*Col1a*, (*Collagen, type I alpha*, OMIM 120150); *Ngn1* (*Neurogenin 1*, OMIM 601726); *Mitf* (Microphthalmia-associated transcription factor, OMIM 156845)] in human NC development (Betters et al., 2010). The NC population migrates to different regions of the mouse embryo from the NT after the epithelial-mesenchymal transition, maintaining its multipotential character until completing differentiation in its final destination (Baggiolini et al., 2015).

To study the ontogeny of the NC, different model organisms, both *in vivo* and *in vitro*, have been used. Several proteins including transcription factors as well as epigenetic modifiers that take part in the specification and differentiation of the NC have been described. The study of transcription factors and of the signaling pathways in which they participate is important to understand the differentiation programs and how these multipotent cells are committed to a specific destination. On the other hand, transcriptome analysis during the development of the NC from specification to migration (Meulemans and Bronner-Fraser, 2004), and a more recent study covering the migration to the differentiation of the NC, show the importance of the interaction between the different transcription factors and the signaling pathways at every stage of NC development (Simões-Costa et al., 2015). However, these authors acknowledge that it is difficult to have a complete global map since only a few transcriptional regulators have been characterized, and little is known about the function of the products of the effector genes acting on NC migration (Betancur et al., 2010; Simões-Costa and Bronner, 2013; Vega-Lopez et al., 2017).

NC and cranial placodes are thought to appear together during the evolution of vertebrates to give rise to specific sensory structures of the head (Northcutt and Gans, 1983; Northcutt, 2005). The components of the sensory nervous system of the head are derived from the NC and from an embryonic cell population developing in close proximity, the cranial sensory placodes (the olfactory, lens, otic, trigeminal, epibranchial and paratympanic placodes). A series of events induce, develop and organize these cell precursors which, through reciprocal interactions with NCCs, build the functional sensory system in vertebrates (Steventon et al., 2014; Singh and Groves, 2016). Migrating NCCs arrive first at the site of ganglia development (i.e., the trigeminal ganglion), but the differentiation of these cells is delayed until the migration and differentiation of the corresponding placodal cells in chicks (Covell and Noden, 1989). Placodal specification and development, as well as its contribution to the assembly of placodal derivatives, is a complex and wide-ranging topic that is beyond the scope of this review. We will focus on discussing the main signaling pathways and relevant transcription factors involved in the specification of cranial NCCs precursors, their differentiation to form CNs and ganglia that are exclusively NC-derived, and the alterations caused by the mutations of certain genes that are important for the neurogenesis of NC derivatives.

## Signaling Pathways Involved in Cranial Neural Crest Development

There are several signaling pathways and transcription factors that are known to regulate NC and CN formation during development. We discuss some important pathways involved in cranial NCCs induction and specification, in close relationship with the cranial ganglia and nerves derived from the NC (Figure 3).

### BMPs

Bone morphogenetic proteins are proteins that control several important steps in the formation and differentiation of the CNS of vertebrates. These proteins act in different regions of the CNS to regulate fate, proliferation and differentiation. After gastrulation, the presence of BMPs and the activation of this signaling pathway are essential for the differentiation of the non-neural ectoderm whereas the inhibition of this pathway is required for the proper formation of the neural plate. It has been proposed that the later activation of BMPs receptors participates in the induction of the NC through a very fine regulation where the presence of BMPs at a specific time will give rise to the NC in mouse and human Embryonic Stem Cells (ESCs) (Figure 3B) (Mizuseki et al., 2003; Leung et al., 2016).

Seminal studies in *Xenopus* have shown that there is an activity gradient of BMPs controlled by their antagonists and that an intermediate level is needed to induce the formation of the NC (LaBonne and Bronner-Fraser, 1998; Marchant et al., 1998; Barth et al., 1999; Tribulo et al., 2003). Thus, the BMP antagonists Chordin (OMIM 603475) and Noggin (OMIM 602991) are expressed in a spatio-temporal manner that influences the formation of the NC. In mouse, at embryonic day (E) 8.0, Noggin is expressed in the neural folds and in the dorsal region after the closure of the NT. The expression of Chordin is low at the level of the neural plate and in the paraxial mesoderm. These antagonists participate in the induction of NC as well as in delamination, but also protect from apoptosis induced by BMP during migration and differentiation of NCCs. Importantly, it was observed that the decrease in the expression of these BMP antagonists alters the PNS derived from the NC and craniofacial skeletal elements. Noggin knockout mice presented all cranial nerves, but the vagus (X) and glossopharyngeal (IX) are disorganized and fused. Double-knockout mice of Noggin and Chordin lack CN and only a structure similar to the trigeminal ganglion (V) is present (Anderson et al., 2006). In the chick embryo, the activity of BMP signaling during the formation of NC precursors is modulated by CKIP/Smurf factors through the regulation of Smad degradation, resulting in intermediate levels of BMP activity required for proper NC formation (Piacentino and Bronner, 2018). In contrast, placode progenitors have differential BMP signaling requirements as they can be specified under low or no BMP signaling (Thiery et al., 2020).

A study of human ESCs (hESC) showed that if BMPs are blocked with Noggin for 24 h on days 0, 1, or 2 of the differentiation protocol, there is a dramatic decrease in the induction of human NCCs. However, if the inhibition is made on day 3, the inhibition is partial, so the participation of BMPs at the beginning of the induction of the NC is very important, while the inhibition of this pathway promoted the expression of neural genes such as *SOX1* (OMIM 602148), *HES5* (OMIM 607348), and *SOX2* (OMIM 184429) (Leung et al., 2016). This protocol produced sensory peripheral neurons, and it will be of interest to investigate if such neurons can contribute to the sensory CN after grafting them in experimental animals, as well as the effect of modulating BMPs on peripheral neuron differentiation. Interestingly, BMP antagonism upregulates these neural stem cell markers, but several reports indicated that Sox1, Hes5, and Sox2 are involved in the suppression of neuronal differentiation by maintaining neural stem and progenitor cells in an undifferentiated state in mammalian cells (Kan et al., 2004; Bani-Yaghoub et al., 2006). The generation of neurons from stem cells depends on the decrease of *Sox1-3* expression caused by proneural proteins. However, if *Sox1-3* target genes were repressed, independently of proneural activity, neural progenitor cells differentiated prematurely, and some neuronal features emerged. These results demonstrate a dual role of proneural proteins in the acquisition of a definitive neuronal fate and indicate that the proneural protein-directed repression of *Sox1-3* expression is a required and irreversible step in the commitment to neuronal differentiation in several species, including mammals (Guillemot, 1999; Farah et al., 2000; Bylund et al., 2003).

BMP4 (OMIM 112262) and Smad proteins have been involved in an interesting mechanism called retrograde signaling in trigeminal ganglia from rats (Ji and Jaffrey, 2012). This mechanism elicits a specific transcriptional response that contributes to the specification of different subpopulations of sensory neurons in the trigeminal ganglia (CN V). As axons from the neurons of trigeminal ganglia grow and extend into their three main peripheral axonal branches (ophthalmic, maxillary and mandibular) that innervate the corresponding regions of the face, they encounter BMP4, which results in a retrograde signal that leads to transport back transcription factors SMAD-1, -5, and -8 from axons to the somata, where nuclear accumulation of the phosphorylated and transcriptionally active Smad forms contributes to neuronal specification and ganglia patterning (Nohe et al., 2004; Ji and Jaffrey, 2012). BDNF (Brain-Derived Neurotrophic Factor, OMIM 113505) signaling was also found to regulate axonal levels of SMAD-1, -5, and -8 in concert with BMP4, for patterning of the trigeminal ganglia (Ji and Jaffrey, 2012).

### Hippo Pathway

Genetic studies have demonstrated that Hippo signaling is crucial in organ size regulation, controlling cell number by modulating cell proliferation and apoptosis processes (Huang et al., 2005). Hippo is a critical factor for proliferation and epithelial-mesenchymal transition during embryonic development and cancer. In the neural tube of the mouse, chicken, and frog, YAP (Yes-Associated Protein, OMIM 606608) is expressed in the ventricular zone progenitor cells and co-localizes with the neural progenitor cell marker Sox2 (Milewski et al., 2004; Cao et al., 2008). It has been observed that the ectopic expression of one of the transcriptional regulators of this pathway, TAZ (Transcriptional Coactivator With PDZ-Binding Motif, OMIM 607392) in mammalian cells, stimulates cell proliferation, reduces the inhibition by contact and promotes the epithelial-mesenchymal transition (Lei et al., 2008).

A relationship between this signaling pathway and the classical NC genes, such as interaction with Pax3 is through TAZ and the phosphoprotein YAP65. These proteins participate as co-activators of Pax3. It has been suggested, using transgenic mice, that Tead2 (TEA Domain Family Member 2, OMIM 601729) is an endogenous activator of Pax3 in NCCs (Milewski et al., 2004). Through expression assays, Pax3 and Yap65 were co-localized in the nucleus of NC progenitors in the dorsal region of the NT. Hippo/TAZ/YAP are critical for Schwann cell proliferation and differentiation in a stage-dependent manner. Nuclear TAZ/YAP complexes activate cell cycle regulators to promote Schwann cell proliferation while directing differentiation regulators in cooperation with Sox10 for myelination in rodents (Deng et al., 2017).

*Neurofibromatosis 2* (*Nf2*, OMIM 101000) is a tumor suppressor that inhibits YAP during dorsal root ganglia (DRG) development. *Merlin*, encoded by the *NF2* tumor-suppressive gene, was identified through genetic studies in mouse embryos and proved to be an important upstream regulator of the Hippo-Yap pathway. Neurofibromatosis is an inherited disease characterized by the development of bilateral Schwann cell tumors originated from CN VIII. Mouse with specific Schwann cell-inactivated *Nf2* alleles developed schwannomas and SC hyperplasia (McClatchey et al., 1998; Giovannini et al., 1999, 2000). Merlin has also been shown to act as a suppressor of mouse neural progenitor proliferation, by inhibiting TAZ/YAP pathway activity (Lavado et al., 2013). The mechanism by which Merlin regulates YAP activity might involve p21 Protein-activated kinase 1 (PAK1, OMIM 602590) activation, which induces phosphorylation of Merlin, thus abrogating its scaffold function for YAP and LATS1/2 (OMIM 603473/604861), and thereby attenuates YAP phosphorylation by LATS1/2 in mouse cells (Sabra et al., 2017); it has been suggested that nuclear export signals of Merlin mediate YAP nuclear export in epithelial mammalian cells (Furukawa et al., 2017).

Hindley and co-workers investigated the role of Hippo/YAP signaling in NC development and neural differentiation. They showed that the activity of YAP promotes an early NC phenotype accompanied by premature migratory behavior, and that Hippo/YAP interacts with RA signaling in hESCs (Hindley et al., 2016). A recent study demonstrates that YAP is necessary for the migration of a premigratory pool of NCCs, since they incorporated YAP signaling into a BMP/Wnt-dependent molecular network responsible for the migration of trunk-level NC in avians (Kumar et al., 2019).

### Notch Signaling

Notch is a family of conserved receptors whose activation is induced by specific ligands, Delta-1 (OMIM 606582), Delta-3 (OMIM 602768), Delta-4 (OMIM 605185), Jagged-1 (OMIM 601920), and Jagged-2 (OMIM 602570), through interaction with four possible receptors (Notch1-4) (Perdigoto and Bardin, 2013). Once the Notch receptors are activated through the cell-cell interaction, proteolytic cuts are carried out resulting in the release of the Notch Intracellular Domain (NICD) (Mumm et al., 2000). NICD translocate to the nucleus and forms a transcriptional complex together with the DNA binding protein CBF1 (C promoter binding factor 1, OMIM 147183). This complex recognizes the specific sequence (C/T)GTGGGAA in its target genes, for example Hes1 (OMIM 139605) (Kageyama et al., 2000).

Notch1 receptor is present during development of the rhomboencephalon at E9.5 in mice, showing strong expression within the hindbrain, including the trigeminal, geniculate, petrosum and nodose placodes, which give rise to CN V, VII, IX and X, respectively, and is also expressed in the otic and olfactory vesicle (Reaume et al., 1992). A study where human induced pluripotent stem cells were induced toward NC differentiation showed that when Notch signaling is blocked using a γ-secretase inhibitor (DAPT) or shRNA for *JAGGED-1*, the genes specifying NC [*DLX5* (*Distal-less homeobox 5*, OMIM 600028), *PAX3, SNAI2, SOX10*, and *TWIST1* (OMIM 601622)] are down-regulated. However, the ectopic expression of NICD1 increased its expression, demonstrating that Notch also participates significantly in NC induction (Noisa et al., 2014). Mead and Yutzey evaluated the function of Notch signaling in murine NC-derived cell lineages *in vivo*. They demonstrated that cell-autonomous Notch has an essential role in proper NCCs migration, proliferation and differentiation, with critical implications in craniofacial, cardiac and neurogenic development (Mead and Yutzey, 2012).

### Sonic Hedgehog

Sonic Hedgehog (Shh) signaling is involved in the correct development of NC and therefore in the generation of its cellular derivatives (Figure 3B). Shh is a member of the family of the secreted Hedgehog proteins: Sonic (*Shh*, OMIM 600725), Indian (*Ihh*, OMIM 600726), and Desert Hedgehog (*Dhh*, OMIM 605423). Shh regulation during NC differentiation is crucial during head and face morphogenesis. Mutant mice and humans lacking Shh present holoprosencephaly and cyclopia due to the lack of separation of the forebrain lobes (Chiang et al., 1996). It is suggested that Shh inhibition maintains *Pax3* expression, so the lack of Shh-mediated regulation for Pax3 induction promotes the constitutive induction of NC, generating the aforementioned phenotypes. A subset of Fox genes regulated by Shh signaling is important during lip morphogenesis in mice. Either Shh addition or *Foxf2* (OMIM 603250) overexpression was shown to be sufficient to induce cranial NCCs proliferation (Everson et al., 2017).

On the other hand, enhanced Shh signaling in mouse, mediated by loss-of-function (*Ptch1^*Wig/Wig*^*) of the Shh receptor Patched1 (*Ptch1*, OMIM 601309), suppressed canonical Wnt signaling in the CN region. This critically affected the survival and migration of cranial NCCs and the development of placodes, as well as the integration between NC and placodes (Kurosaka et al., 2015). *Ptch1^*Wig/Wig*^* mutants exhibited severely disorganized trigeminal (CNV) and facial nerves (CNVII) that did not develop properly and failed to project to their appropriate target tissues (Kurosaka et al., 2015). High levels of Shh signaling have been correlated with Moebius Syndrome, which is characterized by cranial nerve defects including trigeminal, abducens (CNVI) and facial alterations concurrent with other craniofacial defects (Verzijl et al., 2003; Vega-Lopez et al., 2018). NCCs migration is particularly sensitive to Shh levels since in mice lacking Shh, these cells continue their migration beyond the normal position and fuse medially, condensing into a single midline ganglion (Fedtsova et al., 2003). Mutation in the mouse Hedgehog acyltransferase (*Hhat*, OMIM 605743) gene produced hypoplasia and aberrant fusion of cranial ganglia (CN V, VII, IX, and X) and affected NC and placode gene markers expression, suggesting that a regionalized action of the Hedgehog signaling is required for proper cranial ganglia and nerve development and patterning (Dennis et al., 2012). *In vitro* analyses showed that Shh increased the number of cranial NC progenitors, from quail embryos, yielding neural and mesenchymal lineages. Shh can decrease the neural-restricted precursors without affecting survival or proliferation. These data also suggest that the mesenchymal-neural precursor was able to yield both the PNS and superficial skeleton (Calloni et al., 2007).

### Receptor Tyrosine Kinase (RTK) Family

Humans have 58 known RTKs, which fall into 20 subfamilies. A few years ago, a systematic work summarized the contribution of the mouse model to the understanding of the role of a subset of RTKs in regulating the activity of NCCs in development (Fantauzzo and Soriano, 2015). With respect to its downstream signaling, RTKs induce the activation of various pathways, including PLC-γ, PI3K, MAPK, JNK, Shc, Erk, and the JAK/STAT pathways. In this section, we discuss insights pointing to mechanisms of action of some RTK families in relation to the development of the cranial NC that have emerged from recent evidence.

#### Eph Receptors

Ephrin ligands and Eph (erythropoietin-producing human hepatocellular carcinoma) receptors comprise an increasingly well studied family of signaling molecules. Ephrins bind to two families of transmembrane tyrosine kinase receptors, EphA and EphB. While A-type Ephrins preferentially bind to EphA receptors, B-type Ephrins do so to EphB receptors. In *Xenopus*, the streams of NCCs going to the second branchial arch express Ephrin-B2, whereas cells reaching the third arch express EphB1; disruption of Eph-Ephrin signaling results in aberrant migration of NCCs, causing mixing of the streams in the branchial pouches (Smith et al., 1997). Eph receptor functions are best characterized in the mouse nervous system, where they are involved in neuronal development and axon guidance (Wilkinson, 2001; Xu and Henkemeyer, 2012), migration and proliferation (Conover et al., 2000; Holmberg et al., 2006; Jurek et al., 2016) as well as inflammation (Coulthard et al., 2012).

The Ephrin ligand/Eph receptor proteins are widely expressed in embryonic tissues. Eph receptors participate in the development of several NC-derivatives in mouse: teeth and the establishment of tooth nerves (CN V) (Luukko et al., 2005; Stokowski et al., 2007; Arthur et al., 2009; Diercke et al., 2011; Matsumura et al., 2017) and participate in cochlear innervation patterns (Zhou et al., 2011). Eph receptors play a role in mouse segmentation and boundary formation of the developing hindbrain, which results in the formation of rhombomeres (r), which are crucial for the orderly formation of CN and specification of NCCs (Flenniken et al., 1996; Flanagan and Vanderhaeghen, 1998; Merrill et al., 2006; Mellott and Burke, 2008; Klein, 2012). Mouse *EphA5*^–/–^ (OMIM 600004) had only <15% of the normal complement of Gonadotropin-releasing hormone neurons in the brain (Gamble, 2005). This also produced infertility in adult female homozygous GNR23 mice, providing a causal link between Ephrin-related mutations and human hypogonadotropic hypogonadism such as Kallman syndrome. It has been shown through genetic labeling that a fraction of GnRH neurons are derived from NCCs (Forni et al., 2011).

A key step in epigenetic control of expression is gene silencing by hypermethylation of CpG islands present at promoter regions (Nakao, 2001). Both specific enzymes and methyl-CpG-binding proteins (MBPs) play a major role in the epigenetic control of gene expression through the recognition and binding to methylated DNA, as well as by the recruitment of remodeling complexes (Defossez and Stancheva, 2011). During development, EphA5 receptor controls the axonal mapping of retinal ganglion cells in the visual system (Zhou, 1997). Recent findings showed site-specific differences in methylation of CpG islands in the *EphA5* promoter, which could account for the activation or repression of this promoter and might influence the graded *EphA5* expression in the mesencephalic tectum (Petkova et al., 2011). During mouse embryonic development, high levels of EphA5 protein were also found in cranial nerve ganglia V, VIII, X, and XII (Cooper et al., 2009). Therefore, it seems reasonable to speculate that this epigenetic methylation may regulate the neurogenesis of these cranial nerves as it does in the myencephalic region.

#### EGFR/ErbB Receptors

The Epidermal Growth Factor Receptor (EGFR, OMIM 131550) and the related ErbB (v-erb-b2 avian erythroblastic leukemia viral oncogene B) proteins transduce after EGF (OMIM 131530) binding. *ErbB2*^–/–^ (OMIM 164870) mice die around midgestation due to cardiac defects. Cranial ganglia are also morphologically aberrant and these embryos show an altered pattern of ErbB3 (OMIM 190151) staining (Meyer and Birchmeier, 1995; Erickson et al., 1997; Britsch et al., 1998; Garratt et al., 2000). *ErbB3* mutant mice embryos die at a later stage as they have reduced numbers of Schwann cell precursors derived from NCCs and therefore lack cranial ganglia nerves, caused by the death of around 80% of both motor and sensory neurons (Riethmacher et al., 1997). Chick NCCs from the hindbrain and ectodermal cells from placodes, participate in the development of cranial ganglia (D’amico-Martel and Noden, 1983; Le Douarin et al., 1986). A chemical mutagenesis screen in Sox10-reporter mice identified an amino acid substitution in the extracellular portion of ErbB3 that resulted in alterations in homozygotic mutants similar to those reported in *ErbB3* knock-outs (Buac et al., 2008).

*ErbB4* (OMIM 190151) null mouse die at mid-gestation, at E11, due to cardiac defects (Gassmann et al., 1995). In order to overcome this lethality, *ErbB4* mutant mice were engineered to express ErbB4 only in the heart. The embryos survived, but presented aberrant cranial nerve architecture, such as ectopic nerve projections of trigeminal (V) and facial (VII) ganglia (Tidcombe et al., 2003). These results suggested the participation of ErbB4 in the control of NCCs migration and axon extension. ErbB4 (alongside Ephrin) is expressed in r3 while one of its ligands, *Neuregulin 1* (OMIM 142445), is expressed in r2 and r4 (Golding et al., 2000, 2004).

#### FGF Receptors

Fibroblast growth factor signaling is composed of 22 members, although only eighteen FGFs signal via FGF Receptor (FGFR) interactions (FGF1–10 and 16–23). There are seven signaling receptors, encoded by four FGFR genes, FGFR1–4 (Zhang et al., 2006). FGFs exert their cellular effects by interacting with FGFRs, but FGF-FGFR complexes can only be formed in the presence of heparan sulfate (Pellegrini et al., 2000; Schlessinger et al., 2000). FGFRs, a class of RTK, dimerize and undergo transphosphorylation of the kinase domain upon ligand binding. Four signaling pathways can be activated to transduce intracellularly: MAP Kinase (MAPK), PI3K/AKT, PLC-γ, and STAT (Ornitz and Itoh, 2015).

In mice, FGF signaling is necessary for cell survival during the development of tissues, including the embryonic telencephalon and the mid-hindbrain junction (Sato et al., 2004; Zervas et al., 2005; Paek et al., 2009). In zebrafish and chick, FGF3 (OMIM 164950) and FGF8 (OMIM 600483) emanating from r4 are both necessary and sufficient to promote the development of the adjacent r5 and r6 by regulating the expression of transcription factors including Krox20 (Maves et al., 2002; Walshe et al., 2002; Waskiewicz et al., 2002; Wiellette and Sive, 2003; Hernandez et al., 2004; Aragón et al., 2005; Labalette et al., 2011).

Fibroblast growth factor activating FGFR-2(IIIb) (OMIM 176943) at placodal sites (Pirvola et al., 2000), and RA, primarily associated with NC-derived mesenchyme (LaMantia et al., 2000), modulate multiple aspects of sensory neuronal differentiation, including cranial sensory neuron survival, neurogenesis and cranial nerve differentiation. *FGFR-2(IIIb)* knock-in mouse shows severe dysgenesis of the cochleovestibular membranous labyrinth and sensory patches of the vestibulocochlear ganglion (CN VIII) remain small and poorly developed (Pirvola et al., 2000).

MBD1 (Methyl-CpG-binding domain protein 1, OMIM 156535)-null neural stem cells display impaired neurogenesis and increased genomic stability. A possible mechanism is the direct binding of MBD1 to the hypermethylated promoter region of the important neural growth factor *FGF2*. In agreement, MBD1 loss-of-function induces the *FGF2* promoter hypomethylation, thus increasing its expression in mouse adult neural stem cells, which prevents differentiation (Li et al., 2008). Ma et al. showed that *Gadd45b* (Growth arrest and DNA damage-inducible gene 45 beta, OMIM 604948) could induce demethylation in promoters of several genes that participate in mouse neurogenesis, including *Bdnf* (region IX) and *FGF1* (promoter B, OMIM 131220) (Alam et al., 1996). Interestingly, attenuated dendritic growth was found in *Gadd45b* knock-out mice after electro-convulsive treatment, compared to wild-type animals, indicating that Gadd45b is required for DNA demethylation in adult neurogenesis (Ma et al., 2009). Whether or not these mechanisms are shared in NC differentiation to CN is an interesting research topic.

#### PTK7 Receptors

Protein tyrosine kinase 7 (PTK7, OMIM 601890), also named Colon Carcinoma Kinase 4 (CCK4) and Kinase-Like Gene (KLG) in chicken, is the only member of this RTK family (Jung et al., 2002). *PTK7* null mice die perinatally (Lu et al., 2004). Interestingly, Chuzhoi mice, which are homozygous for an ENU-induced splice site mutation in the *PTK7* gene, also die perinatally and similarly to null individuals, exhibit severe neural tube closure defects, have abnormal NCCs distribution and display altered morphology of cranial ganglia and DRG, cardiac outflow tract and ventricular septal defects (Paudyal et al., 2010). PTK7 regulates NC migration via β-Catenin-independent Wnt signaling, and it has been shown that ROR2 (RTK-like orphan, OMIM 602337) is capable of replacing PTK7 function in this process (Podleschny et al., 2015). The human *PTK7* gene has a promoter with 420-bp-long CpG islands (Jung et al., 2002), but epigenetic regulation is unclear at this point.

#### Trk Receptors

Trks (tropomyosin-related kinases) receptors are a subfamily of TRKs activated by neurotrophins (McDonald and Hendrickson, 1993; Murray-Rust et al., 1993). Three types of Trks receptors have been identified during vertebrate development: TrkA (OMIM 191315), TrkB (OMIM 600456) and TrkC (OMIM 191316), activated by NGF (OMIM 162030), BDNF/NT-4 (OMIM 162662) and NT-3 (OMIM 162660), respectively (Hempstead et al., 1991).

The mouse deficiency of NT-3 (Huang et al., 1999), TrkA, TrkB or TrkC (Lewin and Barde, 1996) causes variable loss (39–82%) or decrease of nociceptors and low-threshold mechanoreceptors in the trigeminal ganglion (CN V). TrkB has been found to directly interact with ErbB2 (also known as Her2) for signal transduction in human cells (Choy et al., 2017). Mouse TrkB and p75^*NTR*^ (OMIM 162010) serve as co-receptors of Ephrin-A (Lim et al., 2008; Marler et al., 2008; Barton et al., 2014). Trks have been detected in all classes of PNS neurons with the notable exception of parasympathetic neurons of the ciliary ganglion. With regards to sensory neurons, TrkA is expressed only in DRG and other neural crest-derived ganglia, whereas TrkB and TrkC are expressed to some extent in all sensory ganglia. During embryogenesis, up to 70% of DRG neurons express TrkA but this number declines to around 40% in the adult rat. Co-expression in a single neuron of two members of the Trk family is common, e.g., in adult rat DRG few cells express TrkB alone, while the combinations TrkA + TrkB or TrkB + TrkC are more common (McMahon et al., 1994; Lindsay, 1996). TrkB expression is Ca^2+^ dependent in mouse cortical neurons (Kingsbury et al., 2003), but thyroid hormone T3 down-regulates the expression of TrkB through a negative response element located downstream of its transcription initiation site, during the development of rat brain (Pombo et al., 2000). TrkB was shown to be transcriptionally repressed by Runx3, a Runt domain transcription factor, in mouse and human cells (Inoue et al., 2007).

BDNF and NGF signals emanating from chicken sensory ganglia stimulate cranial motor axon growth (Li et al., 2020). MeCP2 (Methyl-CpG-Binding Protein 2, OMIM 300005) acts with REST/NRSF (Re1-Silencing Transcription factor/Neuron-Restrictive Silencer Factor, OMIM 600571) to recruit Histone Deacetylases, causing a decrease in the expression of BDNF. On the other hand, MeCP2 is released from the BDNF promoter in mouse neurons as a consequence of membrane depolarization, thereby allowing its transcription (Ballas et al., 2005; Zhou et al., 2006). Neuronal activity promotes MeCP2 phosphorylation at specific sites, which differentially changes its binding to gene promoters such as BDNF, a step that is decisive for proper neuronal development and synaptic plasticity in mice (Na and Monteggia, 2011).

#### VEGF Receptors

Vascular endothelial growth factor receptors (VEGFRs) are important in the formation of the vascular system during embryonic development. The mammalian VEGFR are three related type III RTKs known as VEGFR1, VEGFR2, and VEGFR3. These receptors, which bind to VEGF ligands, consist of five glycoproteins referred to as VEGFA, VEGFB, VEGFC, VEGFD and placenta growth factor (PlGF) (Ferrara et al., 2003). The transmembrane protein neuropilin 1 (NRP1, OMIM 602069) is essential for the patterning of the facial nerve (VII) in mouse, as it binds the secreted Semaphorin SEMA3A (OMIM 603961) to guide facial branchiomotor axons in invading the second branchial arch. However, NRP1 can also be activated by the VEGF isoform VEGF164 to control the position of facial branchiomotor neuron cell bodies within the chick hindbrain (Anderson et al., 2003; Schwarz et al., 2004). Cranial NCCs express VEGFR2 and its co-receptor NRP1 as they migrate from the hindbrain at the level of r4 to invade pharyngeal arch 2 in response to chemoattraction by VEGF also in chicken (McLennan et al., 2010).

### Wnt Signaling

Wnts proteins are secreted glycoproteins that participate in a wide variety of cellular processes in development and disease. Binding of Wnts to receptors composed of Frizzled and Lrp5/6 triggers a canonical pathway that results in the stabilization of β-catenin (OMIM 116806), which otherwise is phosphorylated by GSK3β (OMIM 605004) and undergoes constant degradation by the proteasome. Stabilized β-catenin interacts with TCF to activate the expression of target genes (Nusse and Clevers, 2017). Non-canonical signaling pathways are associated with Wnts, namely the Planar cell polarity in Drosophila and the Wnt/Ca^2+^ pathway in vertebrates. The latter involves at least two branches: Ror1/2 activation of Phospholipase C, associated with Wnt binding to Frizzled receptors, produces IP_3_ and DAG, which increases cytoplasmic Ca^2+^ concentrations; the second mechanism is the direct activation of Ror1/2 by Wnts, resulting in increases in cytoplasmic Ca^2+^, which activates Calpain (De, 2011). The interaction of Wnt with other signaling pathways, e.g., with the Smad pathway, has been demonstrated in hESCs (Menendez et al., 2011). An efficient method was described for the generation of NCCs from human pluripotent stem cells through the sustained activation of Wnt signaling combined with low Smad signaling, accomplished by the inhibition of the Activin/Nodal pathway. After 12 days, this constant inhibition of Smad considerably inhibited the formation of CNS Pax6 (OMIM 607108)-positive cells and increased the percentage of cells positive for the low affinity neurotrophin receptor, p75^*NTR*^, which is expressed in the migratory NC (Heuer et al., 1990; Wislet et al., 2018). Within the population of p75 positive cells, authors found cells with intermediate levels of p75, but positive for Pax6; in contrast, the cell population that expresses high levels of p75 was positive for Ap-2α (OMIM 107580), characteristic of NCCs (Menendez et al., 2011). Whether or not a chronic inhibition of Smads has a similar effect *in vivo* remains to be tested.

The activities of genes that influence the morphogenesis of the head are related to Wnt signaling through the expression of Wnt antagonist proteins, the main one being *Dkk1* (OMIM 605189). Loss of expression of *Dkk1* promotes an ectopic activation of Wnt/β-catenin signaling during gastrulation. Using *in vivo* assays, it was demonstrated that Dkk1 and Wnt3a (OMIM 606359) are regulated in a negative feedback loop. In agreement with this, 51% of double heterozygous mice for *Dkk1* and *Wnt3a* showed reduced forebrain while 30% were normal. A small percentage of mice had malformations of eyes and pharyngeal arches as well as defects in the trunk. Therefore, regulation of Wnt signaling participates in the formation of the head but also in several mouse NC derivatives, although there are other pathways and transcription factors involved in the morphogenesis of the head (Lewis et al., 2008).

The canonical Wnt pathway prominently participates in the induction, lineage specification, delamination and differentiation of NC derivatives (Figure 3B). Differentiation into several cell types of the mouse NC is dependent on the sequential activation of Wnt signaling, which indicates that the decision of the cellular differentiation is regulated by the activation state of Wnt/β-Catenin (Hari et al., 2012). *In vitro*, Wnt/β-Catenin signaling centrally participates during differentiation to NC, inducing transcriptional factors that are expressed before factors expressed in neural borders, such *PAX3*, *PAX7*, *MSX1*, and *TFAP2A*. These pre-border transcriptional regulators are GBX2, SP5, ZIC3 and ZEB2 (Leung et al., 2016). In the case of Gbx2 and its role in CN formation, the initial characterization of *Gbx2* mutants in mice demonstrated defects, specifically the absence of the trigeminal nerve (CN V) (Byrd and Meyers, 2005). In addition to the several transcription factors that are important in the induction and specification of NC, there are some proteins, such as Heat Shock Proteins, that participate in these processes. An example of this is the heat shock binding protein 1 (HSBP1). A study in mouse and zebrafish showed that HSBP1 participates in both the pre-implantation status of the blastocyst and the development of the NC. This was demonstrated by the deletion of HSBP1, where its absence promoted a cell arrest or degeneration before reaching the blastocyst stage. With respect to NC, mice deficient in *Hsbp1* showed an increase in the expression of inducers of NC, *Snai2*, *Tfap2*α and *FoxD3*, suggesting that HSBP1 has a potential role in the Wnt pathway (Eroglu et al., 2014). The participation of Heat Shock Proteins in neuronal differentiation to form CN has not been explored yet and, given the importance of Wnt signaling for NC, represents an area of opportunity. Some of the functions associated to molecules in NC development are summarized in Table 2.

**TABLE 2:** Cues required for development of NCCs are NC-derived cranial nerves.

**Molecule (in alphabetical order)**	**Participation in neural crest development**	**Proposed role**	**References**
BMPs	Induction, migration and differentiation	Cell fate decision, epithelial-to-mesenchymal transition, delamination, apoptosis	Nie et al., 2006
β-Catenin	Specification Survival and/or differentiation	Conditional inactivation of β-catenin results in increased apoptosis in mouse cranial NCCs and craniofacial malformations	Brault et al., 2001
Dlx2	Survival and differentiation	Involved in survival of zebrafish cranial NCCs and differentiation of sensory ganglia	Sperber et al., 2008
Endothelin-1 and endothelin A receptor	Induction, migration, maintenance of specification, and target invasion	Required for early development and migration into or within the PA 1-4, also in PA D-V patterning	Clouthier et al., 2000, 2003; Abe et al., 2007; Bonano et al., 2008
EphA4, EphB1, and Ephrin-B2	Migration	Prevent intermingling of third and second arch Xenopus NCCs	Smith et al., 1997
Ephs and Ephrins	Migration	Restricts avian and murine NCCs into streams by inhibiting migration into NCC-free zones	Adams et al., 2001; Davy et al., 2004; Mellott and Burke, 2008
ErbB2, ErbB3, Neuregulin	Migration	Defects in proximal cranial sensory ganglia derived from trigeminal otic placodes and from NCCs; defects in sympathetic neuron migration	Lee K.F. et al., 1995; Meyer and Birchmeier, 1995; Erickson et al., 1997
ErbB4	Migration	Maintains the r3-adjacent NCC-free zone	Golding et al., 2000, 2004
FGF2	Proliferation and differentiation	Depending on the concentration of FGF2, either proliferation is enhanced or cartilage differentiation is induced	Sarkar et al., 2001
FGFR1	Target invasion	Provides a permissive environment for NCC migration into branchial arch 2	Trokovic et al., 2005
Gbx2	Induction and patterning	Establishes regional identity and patterning	Steventon and Mayor, 2012
Hand2	Specification	Neural precursor specification	Hendershot et al., 2007, 2008
Hippo/Yap	Specification, and migration,	Interaction between Hippo/YAP and retinoic acid	Hindley et al., 2016
*Hox* genes	Specification, migration and differentiation	Maintain segmental identity of cranial NCCs through unknown mechanism	Hunt et al., 1991; Trainor and Krumlauf, 2000, 2001; Gavalas et al., 2001; Parker et al., 2018
Indian and Sonic Hedgehog	Specification, migration, differentiation and Survival	Reduction in Sonic hedgehog signaling leads to increased neural tube and NCC death	Ahlgren and Bronner-Fraser, 1999; Jeong et al., 2004; Aguero et al., 2012; Cerrizuela et al., 2018
Kreisler (Mafb)	Patterning, precursors cells specification	Hindbrain patterning	McKay et al., 1997; Manzanares et al., 1999
Krox20 (Erg2)	Patterning, precursors cells specification	Hindbrain patterning	Schneider-Maunoury et al., 1993, 1997; Swiatek and Gridley, 1993; Nieto et al., 1995
Msx1/Msx2	Specification, survival and proliferation	Mouse mutants display impaired cranial NCC patterning, survival and proliferation	Han et al., 2003; Tribulo et al., 2003; Tribulo et al., 2004; Ishii et al., 2005
Neurogenin 1	Neuronal differentiation	Loss of proximal cranial sensory neurons derived from trigeminal otic placodes and from NCC	Ma et al., 1998
Neuropilin-1 and Semaphorin-3A, -3F	Migration	Avian and murine cranial NCCs express neuropilin-1 and are repelled by semaphorin-3A	Eickholt et al., 1999; Osborne et al., 2005; Gammill et al., 2006; Schwarz et al., 2008
Neuropilin-1a, -1b, -2a, -2b and Semaphorin-3Fa, -3Ga	Migration	Restricts zebrafish NCCs into streams by inhibiting migration into NCC-free zones	Yu and Moens, 2005
Neuropilin-1 and VEGF	Target invasion	VEGF attracts Neuropilin-1 expressing NCCs into branchial arch 2	McLennan and Kulesa, 2007; McLennan et al., 2010
Neuropilin-2 and Semaphorin-3F	Trigeminal ganglion formation	Mice with null mutations in either molecule display improperly formed ganglia	Gammill et al., 2006
Notch/Hes	Induction, specification, migration, proliferation and differentiation	Ectodermal cell fate decision	Noisa et al., 2014; Vega-López et al., 2015
Otx2	Induction and patterning	Establishes regional identity and patterning	Hoch et al., 2015
Phox2b	Specification, differentiation	Neuronal phenotype decision	Pattyn et al., 1999
PTK7	Migration	Versatile co-receptor in Wnt signaling	Podleschny et al., 2015
Retinoic Acid	Induction, migration	Mediates the segmental migration of cranial NCCs	Lee Y.M. et al., 1995; Menegola et al., 2004; Dupé and Pellerin, 2009; Simkin et al., 2013
		A-P patterning	
Sox	Induction, migration and differentiation	-*Sox9* and *Sox10*: induction and NC development -*Sox22* is expressed in CNV to CNX and might play a role during the human NC differentiation	Hong and Saint-Jeannet, 2005; Chew and Gallo, 2009 Jay et al., 1997
Zic2	Induction	Ectodermal cell fate decision	Elms et al., 2003

*Abbreviations: A-P, antero-posterior; CN, Cranial Nerve; D-V, dorso-ventral; NCC, neural crest cell; PA, pharyngeal (branchial) arch; r, rhombomere.*

## Relevant Neural Crest-Expressed Transcription Factors Required for Neurogenesis and for the Formation of Cranial Nerves and Ganglia

### bHLH Family

#### Hand2

The basic helix-loop-helix (bHLH) DNA binding protein Hand2 (dHand, Thing-2, Hed, OMIM 602407) is expressed in a subset of NC-derived cells where it participates in various aspects of cell specification, lineage segregation, and cell type-specific gene expression (Hendershot et al., 2007, 2008). Loss of Hand2 results in embryonic lethality by E9.5. In order to study the role of Hand2 in NC, a specific deletion of Hand2 was engineered by crossing floxed Hand2 mice with Wnt1-Cre transgenic mice. Hand2 knock-out in NC-derived cells caused severe effects on development in all NC-derived structures and tissues where Hand2 is expressed. In the autonomic nervous system, conditional interruption of Hand2 function results in a marked and progressive loss of neurons concomitant with a loss of Tyrosine Hydroxylase (TH) expression in mice (Hendershot et al., 2008). There are few studies tackling the importance of Hand2 in NC development and differentiation and none about its importance in CN formation.

#### Hes Family

The *Hes* genes are homologs of the Drosophila *hairy and Enhancer of Split* gene. The Hes family is composed of seven members, Hes4 being absent in the mouse genome. *Hes* genes encode nuclear proteins that repress transcription, either actively or passively (Kageyama and Ohtsuka, 1999). These genes have conserved domains that confer the transcriptional function to all Hes factors. The bHLH domain contains the DNA binding site and the dimerization region. Hes factors can form homo- and heterodimers with Hes-related bHLH repressors, such as Hey factors, Mash1 (OMIM 100790), E47 and Ids. The Orange domain regulates the selection of the bHLH heterodimer, and the WRPW Groucho-binding domain at the C-terminus consists of a tetrapeptide Trp-ArgPro-Trp that represses transcription. This sequence also acts as a polyubiquitination signal for the degradation of Hes by the proteasome (Akazawa et al., 1992; Sasai et al., 1992; Ohsako et al., 1994; Kobayashi and Kageyama, 2014). The Hes transcription factors are essential effectors of Notch signaling that regulate the maintenance of progenitor cells and the time of their differentiation into various tissues and organs (Kageyama and Ohtsuka, 1999). Hes1 (OMIM 139505) is a negative regulator of neural differentiation, since it represses the expression of pro-neural genes such as *Mash1*, *Neurogenin-2* (OMIM 606624) and *Math*. Mice deficient for *Hes1* show a severe neural hypoplasia due to accelerated neural differentiation and the consequent depletion of neural precursor cells (Ishibashi et al., 1995). In agreement with the above, Hatakeyama and co-workers demonstrated that the absence of *Hes1* and *Hes5* caused severe alterations in the size, shape and cytoarchitecture of the mouse CNS. They also found that in *Hes1*;*Hes5* double-mutant mice, the cranial and spinal nerve systems were also severely disorganized, pointing to dysregulation of these NC derivatives (Hatakeyama et al., 2006). These results indicate that Hes1 and Hes5 play an important role in the formation of both CN and spinal nerves.

#### Id Proteins

Id proteins are inhibitors of DNA binding and cell differentiation; four members of this family have been described, Id1-Id4. They are negative regulators of bHLH transcriptional factors that are involved in various processes such as neurogenesis, hematopoiesis, myeloid differentiation, and bone morphogenesis, among others. It has been reported that gene expression of *Id* is present in undifferentiated cells, highly proliferating cells, embryonic cells and cancer cells (Roschger and Cabrele, 2017). One of the Id proteins, Id2 (OMIM 600386), directs the ectodermal precursors to NC commitment and neuronal differentiation. It is expressed in the trunk and in cranial folds, and therefore also in cranial NCCs. The ectopic expression of Id2 in chick promoted a switch of ectodermal cells to NC fate. Overexpression of Id2 increases growth and causes premature neurogenesis in the dorsal region of the NT (Martinsen and Bronner-Fraser, 1998). Conversely, loss of *Id2* in mice caused a decrease in newborn neurons while increasing the number of astrocytes (Havrda et al., 2008). It was recently shown that *Id2a* expression decreased in the forebrain, midbrain and hindbrain as a consequence of blocking Mecp2 expression with a morpholino oligonucleotide. This was consistent with the activation of Notch signaling in such morphants. Mechanistically, in Mecp2 morphants, *her2* (the zebrafish ortholog of mammalian *Hes5*), was upregulated in an Id1-dependent manner (Gao et al., 2015).

#### Neurogenins

In avian and mammalian embryos, the proneural transcription factors Ngn1 and Ngn2 are expressed in NCCs during migration previous to their neuronal differentiation into sensory neurons. In mouse embryos, the functional inactivation of both *Ngn* genes led to a total absence of neurons of the DRG (Ma et al., 1999; Perez et al., 1999). In zebrafish, blocking with a morpholino for *Ngn1* leads to a complete loss of neurons in the cranial ganglia and DRG neurons (Andermann et al., 2002; Cornell and Eisen, 2002). Recently, McGraw et al. (2008) demonstrated in zebrafish that, in the absence of *Ngn1*, the sensory neuron-restricted lineage of NC gives rise only to glial cells.

### Homeodomain Family

#### Hox Transcription Factors and Their Regulators

*Hox* genes play a central role in NC patterning, particularly in the cranial region (Figure 3C). These genes are essential for specifying segmental identity in the developing brain in several vertebrate species. The mechanism responsible for *Hox* genes expression at higher relative levels in specific rhombomeres is independent of the process that establishes the axial expression patterns found in the neural tube. *Hox* genes are organized into four distinct clusters (*Hoxa-Hoxd*) located on different chromosomes in higher vertebrates (McGinnis and Krumlauf, 1992).

It has been long proposed that the *Hox* “collinear expression” is the result of a unidirectional chromatin opening from 3′ to 5′ during development (Lewis, 1978; Duboule and Dollé, 1989; Graham et al., 1989). As a result of collinearity, *Hox* genes expressed in the hindbrain are from paralog groups 1–4. Members from groups 5 to 13 have anterior boundaries of expression which map to the spinal cord (Nolte and Krumlauf, 2007). *Hox* paralog group 1 genes have been suggested to influence early cranial NC development through NCC precursors by interacting with factors in the neural plate border or NC specification modules, although direct gene interactions remain to be determined. The expression of *Hox* paralog groups 2–4 genes in mouse cranial NCCs is modified by Hox auto- or cross-regulation in addition to other inputs from NC transcription factors such as AP-2 in the case of Hoxa2 (Parker et al., 2018).

*Hoxa1* (OMIM 142955) mouse null mutants die at birth from anoxia and exhibit marked reductions in the sizes of r4 and r5, hypoplasia of the inner ear and specifically in CNIII. The embryonic phenotype is characterized by the absence of facial nerve and abducens motor nerve (Lufkin et al., 1991; Chisaka et al., 1992). In agreement, a homozygous truncating mutation of *HOXA1* in humans causes severe congenital cardiovascular malformation, craniofacial and inner-ear defects, as well as brainstem abnormalities (Tischfield et al., 2005; Bosley et al., 2008).

*Hoxb1* (OMIM 142968) loss-of-function mouse mutants exhibit alterations in the molecular markers associated with r4 identity, although no overt changes in the anatomy of the developing hindbrain are present (Goddard et al., 1996; Studer et al., 1996). These and previous results demonstrate that Hoxb1 has a normal role in regulating rhombomere identity, and also participates in controlling migratory properties of motor neurons in the hindbrain. In *Hoxb1* mutant animals, the facial branchiomotor neurons (CNVII) and contralateral vestibular acoustic efferent (CNVIII), which are specific to r4, are incorrectly specified (Goddard et al., 1996; Studer et al., 1996). *Hoxb1* deficiency in mouse also results in facial paralysis due to developmental defects in CNVII, originating from r4 (Figure 3C). In mouse lacking both *Hoxa1* and *Hoxb1* expression, the migration and development of NCCs derived from r4 fail, causing the loss of all second arch derivatives (Rossel and Capecchi, 1999; Arenkiel et al., 2004). These *Hoxa1/Hoxb1* double mutants exhibit a wide range of phenotypes, which are not present in each of individual mutants, demonstrating that specification of r4 cell precursors and patterning of the CN VII-XI strongly requires cooperation between these 2 genes (Gavalas et al., 1998; Studer et al., 1998).

*Hoxa2* (OMIM 604685) is the only member of the *Hox* family expressed in r2; this fact explains why *Hoxa2* null mutations in mouse result in homeotic changes transforming second arch elements of NC origin into first arch derivatives, which was correlated with perinatal lethality. Patterning of the hindbrain rostral region also depends on Hoxa2 activity for the establishment of r2 identity and influencing the migration of trigeminal motor axons (CN V) originated from r2/3. In mutant embryos, this CN V, normally derived from r2/3, migrates caudally to exit the hindbrain from r4, the normal site for facial nerve (CN VII), rather than from r2. *Hoxa2* is required for the maintenance of *EphA4* (OMIM 602188) as its expression results selectively abolished in *Hoxa2* mutants (Rijli et al., 1993). The loss of *Hoxb2* (OMIM 142967) in mouse embryos results in impaired development of the facial nerve, CN VII, affecting its somatic motor component (Bailey et al., 1997).

*Hoxa3* (OMIM 142954) null mutant mice show mesenchymal NCCs defects in the formation of CN IX and also fusions between CN IX and X. In addition, *Hoxa3*^–/–^ mouse are athymic, aparathyroid, and have malformations in cartilage of the throat (Chisaka and Capecchi, 1991; Manley and Capecchi, 1995, 1997). *Hoxb3*^–/–^ (OMIM 142966) embryos revealed similar cranial ganglia defects, but at a lower penetrance than in the *Hoxa3* mutants (Manley and Capecchi, 1997). *Hoxb3*/*Hoxd3* (OMIM 142980) double mutants have a clear increase in the presence of aberrant ganglionic phenotypes in CN IX compared to those reported in the *Hoxb3* single mutant, even though the *Hoxd3*^–/–^ does not show defects in these structures (Manley and Capecchi, 1998).

In conclusion, *Hox* patterning genes are crucial for NC development by interacting with signaling pathways that induce NC, but also to regulate expression of several genes involved in these essential cell and developmental processes. Some studies have shown that Polycomb group proteins are decisive in epigenetic silencing *Hox* genes by promoting changes in the chromatin structure. Dynamic patterns of histone modifications and 3D chromatin organization are also relevant regulators of *Hox* gene expression and function (Boyer et al., 2006; Bracken et al., 2006; Lee et al., 2006; Noordermeer et al., 2011). The transcription factors Krox20 (OMIM 129010) and Kreisler (OMIM 608968), as well as the vitamin A derivative RA are the three main upstream regulators of *Hox* gene expression during hindbrain development.

The transcription factor Krox20 binds to specific DNA sequences located at 5′ flanking region of *Hoxa2*, *Hoxb2*, *Hoxb3*, and *EphA4* genes, to directly control their expression (Lemaire et al., 1988; Nardelli et al., 1991). Targeted mutation of *Krox20* in mouse embryos causes perinatal death and fusions of the trigeminal ganglia with facial and vestibular ganglia as a consequence of alterations on hindbrain patterning and morphogenesis. *Krox20* is expressed in r3 and r5 at E8.0 in mouse embryos (Wilkinson et al., 1989; Schneider-Maunoury et al., 1993, 1997; Swiatek and Gridley, 1993; Nieto et al., 1995).

*Kreisler* expression, first detected at E8.5 in the prospective r5 region and later located in r5 and r6, is sharply downregulated afterward in these rhombomeres (Cordes and Barsh, 1994). Gene expression analyses in *Kreisler* mutant embryos and regulatory regions strongly pointed that this transcription factor could directly control the expression of genes required for inner ear and hindbrain development, in particular *Hoxa3* and *Hoxb3*, which increase its expression in r5 and r6 (McKay et al., 1997; Manzanares et al., 1999). The primary defect in *Kreisler* mutant mouse embryos is an alteration in segmentation at the otic region of the hindbrain, resulting in defective rhombomeres since the borders that normally separate r4, r5, and r6 disappear. Consequently, in r6 important alterations are detected: the normal expression domains of *FGF3* and *CRABP1* (OMIM 180230) are lost, and *Hoxa3* is not upregulated (Frohman et al., 1993). Although *Krox20* expression in the prospective r3 is conserved, it is absent in r5. Similarly, the expression of *Hoxb2*, *Hoxb3*, and *Hoxb4* in r5 are completely abolished. The expression pattern analysis of EphA7 and EphrinB2 indicates that only a single region that would correspond to r5 is absent. Thus, loss-of-function of *Kreisler* causes a segmentation defect which results in the precise loss of r5 patterning; furthermore, although the r6 territory forms, it fails to mature (Manzanares et al., 1999).

RA is a morphogen derived from Vitamin A (retinol) that reaches the cell nucleus after diffusing through cell membranes to act on histone acetylation and mediates transcriptional activation of target genes. RA is another important regulator of NC development. As mentioned earlier, Hox gene expression patterns specify AP identity in the hindbrain and this is transferred to NC migration (Briscoe and Wilkinson, 2004; Simkin et al., 2013). The “collinear pattern” of *Hox* gene expression in the hindbrain is partially dependent on RA control. Cellular retinoid-binding proteins (CRBPs) participate in controlling RA concentration locally, and hence facilitate its function. CRBPs might sequester RA and thus limit its availability to bind nuclear RA receptors (RARs and RXRs) that recognize a particular element on target genes, the RARE sequence (Mangelsdorf et al., 1992; Mangelsdorf and Evans, 1995). Members of the *Hox* family that harbor RAREs include *Hoxa1*, *Hoxb1*, *Hoxa4* (OMIM 142953), *Hoxb4* (OMIM 142965), *Hoxd4* (OMIM 142981), and *Hoxb5* (OMIM 142960) (Kesseland and Gruss, 1991; Langston and Gudas, 1992; Marshall et al., 1992, 1994; Studer et al., 1994; Dupé et al., 1997; Gould et al., 1998; Packer et al., 1998; Pera et al., 1999; Power et al., 1999).

Vitamin A-deficient pregnant rats were produced by feeding dams with low levels of all-trans RA. Such embryos presented loss of CN IX, X, XI, and XII and the associated sensory ganglia IX and X, as well as perturbations in hindbrain segmentation and otic vesicle development (White et al., 2000). These embryos have Hoxb1 protein in the NT, but caudal to the r3/r4 border at a time when its expression should be present only in r4, suggesting that RA is essential for neurogenesis, patterning, and segmentation in the posterior hindbrain. *Neuron navigator 2* (*Nav2*) was first identified as an RA-responsive gene required for RA-mediated neurite outgrowth or survival of CN IX and X (McNeill et al., 2010). *Nav2*^–/–^ mouse embryos showed an overall reduction in neurofilament density in the region of CN V to XII.

It was recently found that YAP (a Hippo signaling transcriptional co-activator, see above) regulates the expression of *Hoxa1* and *Hoxc13* in mouse oral and dental epithelial tissues as well as in embryonic and adult epidermal tissue (human keratinocytes) (Liu et al., 2015). Since *Yap* transcript was detected in the rhombencephalon and dorsal NT and also in NCCs that migrate from the dorsal region of the NT to the pharyngeal arches, Yap could regulate the activity of the *Hoxa1* gene expression in the hindbrain.

#### Msx Family

The muscle segment-related homeobox (*Msx*) genes belong to the homeodomain family. These genes code for transcriptional factors with repressor activity. Proteins with homeodomains have various functions during embryonic development, from the formation of expression patterns to more specific functions such as differentiation toward a specific cell type (Catron et al., 1995). *Msx* genes are expressed in a range of vertebrate-specific tissues including NC, cranial sensory placodes, bones, and teeth (Davidson, 1995).

In vertebrates, there are three members of this family, *Msx1-3*; *Msx1*, and *Msx2* being the best characterized ones with respect to their expression pattern and biochemical properties (Bendall and Abate-Shen, 2000). *Msx1* and *Msx2* are expressed in various regions of the mouse embryo such as the NT, in the primordial limbs and in derivatives of the cranial NC (Catron et al., 1996). The expression of *Msx1* and *Msx2* marks the area from which the cranial NC will migrate. *Msx* genes participate in the early specification of NCCs and in the control of apoptotic process under the control of BMP signaling (Tribulo et al., 2003, Tribulo et al., 2004; Ishii et al., 2005).

#### Pax Family

*Pax* genes, which encode transcription factors that contain a highly conserved DNA binding domain called PD, can be considered as the broad regulators of gene expression since they can repress pluripotency genes such as *Oct4*, *Nanog* and *Myc*, or induce the expression of genes involved in the differentiation of NC such as *Snail1* and *FoxD3*. There are nine *Pax* genes (*Pax1*-*Pax9*), which have been characterized in mammals. There is a great diversity of studies on *Pax* genes in the early specification of cell fate and in the morphogenesis of various tissues and organs. The important participation of *Pax* genes in NC induction is discussed next. Pax3 participates in the early ontogenesis of the NT and NC; it is expressed in pre-migratory NCCs. The loss of Pax3 generates severe defects in embryonic development, leading to embryonic death (Goulding et al., 1991). A study in mouse evaluated the participation of the Wnt signaling pathway in the regulation of Pax3. It was demonstrated that the Wnt pathway induces expression of Pax3 indirectly, using Cdx1 as an intermediary that binds the PD domain of Pax3 (Sanchez-Ferras et al., 2012).

On the other hand, transcriptional enhancers are primary determinants of the specific gene expression of a cell type. Recently, an NC enhancer-2 (NCE2) in the 5′ region of *Pax3* was identified as a *cis* regulatory element that is dependent on Cdx as a cofactor. *Pax3* and *Zic2* are expressed in the dorsal region of the NT when it closes. Therefore, the inductive Cdx-Zic2 interaction is integrated by NCE2, allowing the specific binding of the neural transcription factor Sox2 (Buecker and Wysocka, 2012; Sanchez-Ferras et al., 2014). This shows that not only NCE2, but also the transcription factor Zic 2 participate in the regulation of Pax3. Such data suggests that Zic2 is involved in NC induction as an activator of Pax3-NCE2 and as a Cdx co-factor. Mouse *Pax3* mutants (Sp and Sp^*d*^ alleles) additionally exhibit malformations of ganglia of the PNS. The importance of Pax3 in the development of NC-derived structures has been shown, especially with respect to cranial ganglia and nerves. In the homozygous state, Sp and Sp^*d*^ alleles impair the development of the trigeminal (CN V), superior (CN IX), and jugular (CN X) ganglia, suggesting that the function of Pax3 is crucial for NC migration and proliferation, as well as for its differentiation into neurons capable of sending out axons (Tremblay et al., 1995). In *Xenopus* and zebrafish embryos, *Pax3* has been proposed as a key player in the gene regulatory network as a neural plate border specifier controlling early specification of NCC (Hong and Saint-Jeannet, 2007; Minchin and Hughes, 2008; Milet et al., 2013).

Another crucial *Pax* gene in NC formation is *Pax7*, which has been described as necessary for NC development in birds. *Pax7* is required for the expression of NC markers such as *Sox9, Snai2*, *HNK1* and *Sox10* (Basch and Bronner-Fraser, 2006). In human embryos, *Pax7* is expressed in the dorsal NT and in cells of the migratory NC at early stages. In mouse, *Pax7* is expressed in the rostral region, which includes a subpopulation of presumptive NC precursors. Pax7 contributes more to the formation of cranial lineages than to the cardiac or trunk regions. The expression of Pax7 is extensive, since it is detected in mesencephalon, rhombencephalon, dorsal NT, fronto-nasal region and NCCs that migrate from the dorsal region of the NT to the pharyngeal arches (Betters et al., 2010; Murdoch et al., 2012). A mutation of Pax7 (isoform 3) was recently found in patients, causing a phenotype of neurodevelopmental delay during development and promoting microcephaly, irritability and self-mutilation among others symptoms (Proskorovski-Ohayon et al., 2017). Therefore, *Pax7* is a crucial gene in the induction of NC and in its migration.

#### Phox2b

Paired-like Homeobox 2b (Phox2b, OMIM 603851) is a transcription factor known to play a key role in the development of the autonomic nervous system. *Phox2b* is expressed in differentiating neurons of the mouse central and PNS as well as in motor nuclei of the hindbrain. *Phox2a* (OMIM 602753) and *Phox2b* are co-expressed at multiple sites, suggesting a broader role for *Phox2* genes in the specification of autonomic neurons and cranial motor nuclei. The co-expression of these Phox proteins at various sites suggested positive crosstalk (Pattyn et al., 1997). *Mash1* has been demonstrated to control the expression of *Phox2a* (but not of *Phox2b*) in autonomic ganglionic precursors and NC-stem cells (Lo et al., 1998), while *Phox2b* is required for the maintenance but not for the induction of *Mash1* expression (Pattyn et al., 1999). Epistatic analyses have shown that, in cranial ganglia development, *Phox2b* is a downstream effector of *Phox2a* (Pattyn et al., 1999). The mutant *Phox2b*^*LacZ/LacZ*^ mouse showed atrophic cranial ganglia formation that correlated with increased apoptotic cell death and decreased *Ret* and DBH expression in ganglionic anlages (Pattyn et al., 1999). The effect of the *Phox2b* null mutation on cranial ganglia cells was a phenotypical change on their molecular transcriptional signature to *Tlx3^+^/Islet^+^/Phox2b^–^/Phox2a^–^/Brn3a^+^* profile, which means that the sensory neurons present in the cranial nerves VII, IX, and X change to a somatic sensory neuron-like, thus highlighting the role of Phox2b as a molecular switch that commands the somatic-to-visceral phenotype in the cranial sensory genetic cascade (D’Autréaux et al., 2011). Many cranial nerve-associated NCCs co-expressed the pan-autonomic determinant Phox2b and markers of Schwann cell precursors. Such cranial NCC precursors are the source of parasympathetic neurons during normal development (Espinosa-Medina et al., 2014). In humans, PHOX2B over-expression has been linked to the formation of tumors arising from the sympathetic nervous system such as neuroblastomas. Heterozygous *PHOX2B* mutations cause Congenital Central Hypoventilation Syndrome, a life-threatening neurocristopathy characterized by the defective autonomic control of breathing and involving altered CO_2_/H^+^ chemosensivity (Cardani et al., 2018; Vega-Lopez et al., 2018).

### Otx Genes

*Otx1* and *Otx2* genes are the mouse cognates of the *Drosophila* head gap genes. Orthologs have also been identified in human, chick, *Xenopus* and zebrafish. *Otx2* may act as a key head organizer during the primitive streak stage. At subsequent neurula to pharyngula stages, those genes participate in the patterning of the forebrain and midbrain. The haplo-insufficiency mutation of *Otx2* in the mouse affects the mandible and pre-mandibular skull elements, as well as the ophthalmic branch of the trigeminal nerve, and the differentiation of mesencephalic trigeminal neurons, all of which correspond to derivatives originated from mesencephalic NC (Puelles and Rubenstein, 1993; Matsuo et al., 1995). In chick and *Xenopus* embryos, *Otx2* establishes cross-regulatory interactions with *Gbx2* during the early specification of placodal precursors; by mutual repression, both genes pattern the territory, segregating trigeminal progenitors (Steventon et al., 2012). Additionally, *Gbx2* is expressed early in the preplacodal region of *Xenopus* embryos and is required for NCCs formation as an effector of Wnt signaling (Li et al., 2009).

### Sox Family

The proteins encoded by the *Sox* genes belong to the superfamily of the High Mobility Group transcriptional factors that bind to the DNA sequence (A/T)(A/T)CAA(A/T)G. They have a DNA binding domain of 80 amino acids. Based on phylogenetic analyses of their domains, *Sox* genes are divided into subgroups A-H in mouse (Bowles et al., 2000). Some are transcriptional activators, others are repressors, and a third group lacks the transactivation domain.

The subgroup of *SoxE* genes (*Sox8, Sox9* and *Sox10*) has a prominent participation in NC differentiation. In mouse, *Sox9* and *Sox10* are among the first expressed genes in the NC progenitors overlapping with *FoxD3* (Hong and Saint-Jeannet, 2005). A study showed that the defects in the expression of this subgroup affects many lineages of the NC, so these genes are important regulators in the formation of this multipotent population (Kelsh, 2006). However, it is not known if *SoxE* genes are also involved in NC induction in the mouse. Knock-out mice for *Sox9* show expression of *Snai1* in the NC; nevertheless, these cells undergo apoptosis either before or immediately after migrating, which suggests that Sox9 participates in the epithelial-mesenchymal transition, before delamination (Cheung et al., 2005).

Sox10 is a protein that participates in maintaining multipotency in NCCs; it also contributes to proliferation and inhibits differentiation, so this transcriptional factor is expressed in the pre-migratory progenitor cells of the NC and its expression decreases at the beginning of the differentiation process (Kim et al., 2003). Several studies have shown that Sox10 controls the fate of the NC by activating critical genes for the differentiation of different cell types such as melanocytes, Schwann cells, autonomic and sensory neurons in different species (*Mitfa*, *ErbB3*, *Phox2b*, *Mash1*, and *Ngn1*, respectively) (Britsch et al., 2001; Elworthy, 2003; Kim et al., 2003; Elworthy et al., 2005; Kelsh, 2006). Sox10 is regulated by post-translational modifications. For example, changes in the state of SUMOylation affect its function, because it regulates interactions with different proteins and promotes the activation of different genes. *Sox10* expression can be regulated by multiple enhancer elements such as U3, known as MCS4. The stimulation of the U3 enhancer activity promoted *Sox10* transcription, which had a synergistic activity with other transcriptional factors involved in NC development, including Pax3, FoxD3, AP-2α, Krox20, and Sox2 (Taylor and LaBonne, 2005). Sox10 can be self-regulated as well as regulated by synergistic interactions during NC development (Wahlbuhl et al., 2012).

### Zic Family

*Zic* genes are transcription factors with zinc fingers that contribute to different processes during embryonic development (Aruga et al., 1998). It has been proposed that Zic1-3 participate in lateral segmentation, NC induction and inhibition of neurogenesis (Nagai et al., 1997; Nakata et al., 1997). This gene family consists of five members, Zic1-Zic5 in the mouse. *Zic* genes are co-expressed in some cells during embryonic development, which gives the opportunity for heterogeneous protein-protein interactions and/or functional redundancy among family members. *Zic2* (OMIM 603073) is expressed in the cells of the inner mass of the blastocyst and is required for the synchronization of neurulation. *Zic2* mutants showed delayed production and decreased numbers of NCCs. *Zic2* is also necessary for the formation of r3 and r5 and participates in the normal pattern of the mouse rhombencephalon (Elms et al., 2003).

Mouse homozygous mutants of *Zic1* exhibit ataxia during development and die within the first month after birth. These mutants also show a hypoblastic cerebellum and absence of the anterior lobe (Elms et al., 2003). In *Zic* mutants, the expression of *Msx1* in the region of the dorsal NT was not altered; however, its expression was lost in this region when the NT was closing, suggesting that signals from the floor plate are required for the maintenance of dorsal expression (Sanchez-Ferras et al., 2014). *Zic5* (OMIM 617896) is expressed in the dorsal part of the NT and its mutation in humans produces holoprosencephalia, a severe brain malformation. A decrease in Zic5 promotes insufficient NT closure in the rostral-most part, which was also observed with Zic2 (Inoue et al., 2004). *Zic2* mutant embryos showed affected CN V, VII and VII (Elms et al., 2003).

Deficient *Zic5* mice show malformations of the facial bones derived from the NC, mainly the mandible, due to decreased generation of NCCs. During embryonic stages, there were also delays in the development of the first branchial arch and extension of the trigeminal and facial nerves. On the other hand, deletion of *Zic2* promotes congenital malformation of the brain and digits in humans (Inoue et al., 2004). Cranial NCCs are also known to contribute to the development of the PNS. In both mutants, a reduction in the axonal projections from the trigeminal and facial ganglions was reported. These findings suggest that cephalic NC derivatives are selectively affected in these mutants (Inoue et al., 2004).

## Conclusion

Some of the most relevant pathways and genes involved in CN formation are represented in Figure 3 and Table 2. Gene regulation during embryonic development as well as during induction, specification, delamination, migration, survival and differentiation of the NC is a very complex process that leads to a strict expression of genetic information. A remarkable conservation of many genes, signals and mechanisms between different vertebrate organisms, but also its repeated use at different places and times in NC development and cranial nerve/ganglia formation, contributes to the complexity of these processes. Adequate transduction of the signals is equally important for the development and differentiation of each of the cell types derived from the NC. The integration of knowledge from the various studies on such signaling pathways and the different types of proteins that participate in the sequential processes as well as their post-translational modifications will lead to a better understanding of neurogenesis and cranial nerve formation. The microenvironment in which these cells develop is of great importance in order to understand the mechanisms involved in proper NC induction and CN development.

## Author Contributions

All authors listed have made substantial, direct and intellectual contribution to the work and approved it for publication. KM-M did molecular neuroscience data, conception and design, compiling and summarizing articles, manuscript writing, and final approval of the manuscript. GV-L did cell and developmental biology data, conception and design, compiling and summarizing articles, figures elaboration, manuscript writing, and final approval of the manuscript. MA involved in financial support, cell and developmental biology data, conception and design, compiling and summarizing articles, manuscript writing, and final approval of manuscript. IV involved in financial support, molecular neuroscience data, conception design, correction of style, manuscript writing, and final approval of the manuscript.

## Conflict of Interest

The authors declare that the research was conducted in the absence of any commercial or financial relationships that could be construed as a potential conflict of interest.

## Abbreviations

BMP: bone morphogenetic proteins
CN: cranial nerve
CNS: central nervous system
DRG: dorsal root ganglia
FP: floor plate
hESCs: human embryonic stem cells
Msx: Muscle segment-related homeobox
NC: neural crest
NCCs: neural crest cells
NT: neural tube
PA: pharyngeal arches
Pax: Paired box
PNS: peripheral nervous system
r: rhombomere
RA: retinoic acid
RTK: receptor tyrosine kinase
Shh: Sonic hedgehog
Sox: Sry-box
VSMC: vascular smooth muscle cells
Wnt: Wingless and Int-1
WRPW: Trp-Arg-Pro-Trp
Zic: Zinc finger protein of cerebellum.
